# Effect of *Morinda citrifolia* (Noni)-Enriched Diet on Hepatic Heat Shock Protein and Lipid Metabolism-Related Genes in Heat Stressed Broiler Chickens

**DOI:** 10.3389/fphys.2017.00919

**Published:** 2017-11-27

**Authors:** Joshua Flees, Hossein Rajaei-Sharifabadi, Elizabeth Greene, Lesleigh Beer, Billy M. Hargis, Laura Ellestad, Tom Porter, Annie Donoghue, Walter G. Bottje, Sami Dridi

**Affiliations:** ^1^Center of Excellence for Poultry Science, University of Arkansas, Fayetteville, AR, United States; ^2^Department of Animal and Avian Sciences, University of Maryland, College Park, MD, United States; ^3^USDA, Agricultural Research Service, Fayetteville, AR, United States

**Keywords:** heat stress, lipogenesis, lipolysis, noni, quercetin, chicken, liver

## Abstract

Heat stress (HS) has been reported to alter fat deposition in broilers, however the underlying molecular mechanisms are not well-defined. The objectives of the current study were, therefore: (1) to determine the effects of acute (2 h) and chronic (3 weeks) HS on the expression of key molecular signatures involved in hepatic lipogenic and lipolytic programs, and (2) to assess if diet supplementation with dried Noni medicinal plant (0.2% of the diet) modulates these effects. Broilers (480 males, 1 d) were randomly assigned to 12 environmental chambers, subjected to two environmental conditions (heat stress, HS, 35°C vs. thermoneutral condition, TN, 24°C) and fed two diets (control vs. Noni) in a 2 × 2 factorial design. Feed intake and body weights were recorded, and blood and liver samples were collected at 2 h and 3 weeks post-heat exposure. HS depressed feed intake, reduced body weight, and up regulated the hepatic expression of heat shock protein HSP60, HSP70, HSP90 as well as key lipogenic proteins (fatty acid synthase, FASN; acetyl co-A carboxylase alpha, ACCα and ATP citrate lyase, ACLY). HS down regulated the hepatic expression of lipoprotein lipase (LPL) and hepatic triacylglycerol lipase (LIPC), but up-regulated ATGL. Although it did not affect growth performance, Noni supplementation regulated the hepatic expression of lipogenic proteins in a time- and gene-specific manner. Prior to HS, Noni increased ACLY and FASN in the acute and chronic experimental conditions, respectively. During acute HS, Noni increased ACCα, but reduced FASN and ACLY expression. Under chronic HS, Noni up regulated ACCα and FASN but it down regulated ACLY. *In vitro* studies, using chicken hepatocyte cell lines, showed that HS down-regulated the expression of ACCα, FASN, and ACLY. Treatment with quercetin, one bioactive ingredient in Noni, up-regulated the expression of ACCα, FASN, and ACLY under TN conditions, but it appeared to down-regulate ACCα and increase ACLY levels under HS exposure. In conclusion, our findings indicate that HS induces hepatic lipogenesis in chickens and this effect is probably mediated via HSPs. The modulation of hepatic HSP expression suggest also that Noni might be involved in modulating the stress response in chicken liver.

## Introduction

Driven by economic demands and consumer preference for low fat and high protein sources, commercial broiler chickens have been intensively selected for high growth rate and high breast yield (Griffin and Goddard, 1994). Poultry meat and egg production has seen the largest increase during past decades and supports the livelihoods and food security of billions of people worldwide. However, this spectacular progress is also accompanied by several undesirable changes including hyperphagia (Denbow, 1994), metabolic disorders (Julian, 1998; Velleman, 2015), and hypersensitivity to high environmental temperature due to high metabolic activity and lack of sweat glands (Settar et al., 1999; Huang et al., 2015; Velleman, 2015).

Heat stress (HS) is detrimental to poultry production from its strong adverse effects on feed intake, growth, meat yield, welfare, and mortality (Dale and Fuller, 1980; Cahaner and Leenstra, 1992; Deeb and Cahaner, 2002; Deeb et al., 2002). HS resulted in an estimated total annual economic loss to the U.S. poultry industry of more than one-hundred million dollars and, therefore, represents a serious financial burden (St-Pierre et al., 2003). At the animal level, its adverse effects can range from discomfort (mild stress) to multiple organ damage and, under extreme conditions (sever stress), leads to mortality following spiraling hyperthermia. At the cellular level, HS induces many alterations such as, protein misfolding and aggregation (Ruan et al., 2017), cell cycle arrest (Trotter et al., 2001), redox state (Keyse and Emslie, 1992), and transcription modulation (Sanchez et al., 1994). Depending on the type, severity, and duration of the stress, cells can develop highly efficient stress response and protein quality control systems to ensure their survival or activate stress signaling cascades that proceed into cell-death pathways. These responses are controlled by a complex molecular network that is still not completely defined particularly in avian species. At the molecular level, a general rapid response to HS is increased synthesis of heat shock proteins (HSPs). Based on their monomeric molecular size, these ubiquitously expressed chaperones are classified into about six families, with HSP70 and HSP90 being the most extensively studied. Besides their classical roles as molecular chaperones and housekeepers (folding/unfolding, assembly/disassembly), HSPs are now understood to play a pivotal role in many cellular processes including transport and trafficking, protein degradation, and cell signaling (Drew et al., 2014). In turn, these cellular alterations induced by HS can lead to various neuroendocrine, physiological, and immunological adaptations including modulation of lipid and glucose metabolism (Sanz Fernandez et al., 2015; Victoria Sanz Fernandez et al., 2015).

The liver is a highly metabolic tissue that plays a vital role in digestion (bile production), metabolism, immunity, and storage of nutrients as well as detoxification. It has been shown that the liver is responsive and susceptible to HS (Lin et al., 2006; Tang et al., 2015) and that HS alters the hepatic lipid metabolism (Faylon et al., 2015; Dalvi et al., 2017; Jastrebski et al., 2017). Drinking *Morinda citrifolia* (Noni) juice has been shown to enhance hepatic antioxidant capacity, improve lipid homeostasis and protect the liver from environmental and chemical stressors (Wang et al., 2008a,b; Chang et al., 2013; Lin et al., 2017). The botanical name of this *Rubiaceae* plant is originally derived from the two Latin words “morus” ascribing to mulberry, and “indicus” imputing to Indian. It is known as *Noni* in Hawaii, *mulberry* in India, *mengkudu* in Malaysia, *nhaut* in Southeast Asia, *painkiller bush* or *cheese fruit* in Caribbean, *Canary Wood* in Australia, and many other names in different countries (for review see Potterat and Hamburger, 2007). This medicinal plant has been widely used in human nutrition and health (Issell et al., 2009). Due to the recent concerns in the sub-therapeutic use of antibiotics in food animal industries, Noni fruits and leaves have also gained considerable popularity in animal nutrition (Brooks et al., 2009; Sunder and Kundu, 2015; Sunder et al., 2015). However, the effect of Noni on chicken liver metabolism under control and HS conditions is not known.

As chicken liver is the main site for *de novo* fatty acid synthesis (lipogenesis) and also a site for fat storage, and as there is a subtle balance between hepatic lipogenesis and lipolysis, we aimed to determine in the present study the effects of Noni-enriched diet on growth performance, circulating metabolite and hormone levels as well as on the expression of hepatic lipogenesis- and lipolysis-related genes in broiler chickens exposed to acute and chronic HS.

## Materials and methods

### Animals

One-day-old Cobb 500 by-product male chicks (480 total) were randomly assigned into 12 environmentally controlled chambers for rearing. Each chamber was composed of 2 pens (20 bird/pen) with their own feeder and water line. Each pen was given one of two diets: starter corn-soy based diet (Control, 3.9 Mcal ME Kg^−1^ and 21% CP), or control diet supplemented with 0.2% dried Noni plant (Noni, 3.9 Mcal ME Kg^−1^, 19.8% CP), a commonly incorporated levels by poultry producers in tropical regions (Sunder and Kundu, 2016). Chicks were given *ad libitum* access to feed and clean water. The ambient temperature was reduced gradually from 32°C to 24°C with relative humidity at 55 ± 5% for 21 days. On d 22, birds were allocated into two environmental conditions (thermoneutral TN, 24°C vs. heat stress HS, 35°C for 2 h or 3 weeks) and received two finisher diets (control, 3.9 Mcal ME kg^−1^ and 16.4% CP vs. Noni, 3.9 Mcal ME kg^−1^ and 16.8% CP) in a 2 × 2 factorial design. The day before the experiment, the chickens were equipped with a Thermochron temperature logger (iButton, DS1922L, Maxim, CA) for continuous monitoring of core body temperature. The environmental temperature and relative humidity (RH) were also continuously recorded in each chamber. Birds were humanely euthanized by cervical dislocation after acute (2 h) and chronic (3 week) HS. Blood samples were collected aseptically from wing veins using vacutainers with PST gel and lithium heparin (BD, NJ) at each time point and plasma was separated after centrifugation (1,500 g, 10 min, 4°C) and stored at −20°C for later analyses of circulating metabolites. Liver samples (*n* = 8/group per time point) were quickly isolated, snap frozen in liquid nitrogen, and stored at −80°C for subsequent gene and protein expression analyses. All experimental procedures involving animals used in this study were conducted in accordance with the recommendations in the guide for the care and use of laboratory animals of the National Institutes of Health and the protocol was reviewed and approved by the University of Arkansas Animal Care and Use Committee.

### Cell culture

Spontaneous immortalized chicken embryo liver cells (sim-CEL, Piekarski et al., 2014) were grown in Waymouth's media (Life Technology, Waltham, MA) supplemented with 10% fetal bovine serum (Life Technologies, Waltham, MA), and 1% penicillin/streptomycin (Biobasic, Amherst, NY) at 37°C under a humidified atmosphere of 5% CO_2_ and 95% air. At 80–90% confluency, cells were exposed to two environmental conditions (45°C vs. 37°C) and two treatments (50 μM quercetin, QCT vs. control) for 4 h. The dose of QCT was selected based on pilot and previous studies (Dok-Go et al., 2003; Martirosyan et al., 2016; Wang et al., 2017)

### RNA isolation, reverse transcription (RT), and quantitative real-time PCR

Total RNA isolation from liver samples and sim-CEL cells was performed using Trizol® reagent (ThermoFisher Scientific, Rockford, IL) according to manufacturer's recommendations. RNA integrity and quality was assessed using 1% agarose gel electrophoresis and RNA concentrations and purity were determined for each sample by Take 3 Micro-Volume Plate using Synergy HT multi-mode micro plate reader (BioTek,Winooski, VT). The RNA samples were DNase treated and 1 μg RNA was reverse transcribed using qScript cDNA Synthesis Kit (Quanta Biosciences, Gaithersburg, MD) as we previously described (Rajaei-Sharifabadi H. et al., 2017). Real-time quantitative PCR (Applied Biosystems 7500 Real-Time PCR system) was performed as described previously (Rajaei-Sharifabadi H. et al., 2017) using 5 μL of 10X diluted cDNA, 0.5 μM of each forward and reverse specific primer, and SYBR Green Master Mix (ThermoFisher Scientific, Rockford, IL) in a total 20 μL reaction. Oligonucleotide primers used for chicken ATP citrate lyase (ACLY), acetyl-CoA carboxylase alpha (ACCα), fatty acid synthase (FASN), stearoyl-CoA desaturase (SCD-1), malic enzyme (ME), sterol regulatory element-binding protein 1 and 2 (SREBP-1/2), SREBP Cleavage-Activating Protein (SCAP), insulin induced gene 2 (INSIG2), lipoprotein lipase (LPL), hepatic triglyceride lipase (LIPC), adipose triglyceride lipase (ATGL), fatty acid translocase (CD36), peroxisome proliferator-activated receptor alpha and gamma (PPARα/γ), and ribosomal 18S as housekeeping gene were summarized in Table 1. The qPCR cycling conditions were the same as described in Lassiter et al. (2015). Relative expression of target genes was determined by the 2^−ΔΔCt^ method (Schmittgen and Livak, 2008) and the group with control diet under thermoneutral condition was used as calibrator.

**Table 1:** Oligonucleotide real-time qPCR primers.

**Gene**	**Accession number^a^**	**Primer sequence (5′ → 3′)**	**Orientation**	**Product size (bp)**
ACCα	NM_205505	CAGGTATCGCATCACTATAGGTAACAA	Forward	74
		GTGAGCGCAGAATAGAAGGATCA	Reverse	
ACLY	NM_001030540	CTTTTAAGGGCATTGTTAGAGCAAT	Forward	65
		CCTCACCTCGTGCTCTTTCAG	Reverse	
FASN	J03860	ACTGTGGGCTCCAAATCTTCA	Forward	70
		CAAGGAGCCATCGTGTAAAGC	Reverse	
SCD-1	NM_204890	CAATGCCACCTGGCTAGTGA	Forward	52
		CGGCCGATTGCCAAAC	Reverse	
ME	AF408407	AGATGAAGCTGTCAAAAGGATATGG	Forward	62
		CACGCCCCTTCACTATCGA	Reverse	
SREBP-1	AY029224	CATCCATCAACGACAAGATCGT	Forward	82
		CTCAGGATCGCCGACTTGTT	Reverse	
SREBP-2	AJ414379	GCCTCTGATTCGGGATCACA	Forward	63
		GCTTCCTGGCTCTGAATCAATG	Reverse	
SCAP	XM_001231539	TGGCCCAGAGACTCATCATG	Forward	67
		GCAGGATCCGTATAAACCAGGAT	Reverse	
INSIG2	NM_001030966	CAGCGCTAAAGTGGATTTTGC	Forward	65
		CAATTGACAGGGCTGCTAACG	Reverse	
PPARα	AF163809	CAAACCAACCATCCTGACGAT	Forward	64
		GGAGGTCAGCCATTTTTTGGA	Reverse	
PPARγ	NM_001001460	CACTGCAGGAACAGAACAAAGAA	Forward	67
		TCCACAGAGCGAAACTGACATC	Reverse	
LPL	NM_205282	GACAGCTTGGCACAGTGCAA	Forward	62
		CACCCATGGATCACCACAAA	Reverse	
LIPC	XM_425067	GGTCTCAGTGTTGGCATCAAAC	Forward	72
		AGGCTGAAAGGTGCCTCCAT	Reverse	
ATGL	EU240627	GCCTCTGCGTAGGCCATGT	Forward	60
		GCAGCCGGCGAAGGA	Reverse	
CD36	NM_001030731	ACTGCGCTTCTTCTCCTCTGA	Forward	68
		TCACGGTCTTACTGGTCTGGTAAA	Reverse	
18S	AF173612	TCCCCTCCCGTTACTTGGAT	Forward	60
		GCGCTCGTCGGCATGTA	Reverse	

a *Accession number refer to Genbank (NCBI). ACCα, acetyl-CoA carboxylase alpha; ACLY, ATP citrate lyase; ATGL, adipose triglyceride lipase; CD36, fatty acid translocase; FASN, fatty acid synthase; INSIG2, insulin-induced gene 2; LIPC, hepatic triacylglycerol lipase; LPL, lipoprotein lipase; ME, malic enzyme; PPARα/δ, peroxisome proliferator activated receptor α/δ; SCAP, sterol regulatory element-binding protein cleavage-activating protein; SCD-1, stearoyl-CoA desaturase 1; SREBP-1/2, sterol regulatory element-binding protein 1/2*.

### Protein isolation and western blot analysis

Liver tissues and sim-CEL cells were homogenized in lysis buffer as previously described (Lassiter et al., 2015). Total protein concentrations were determined using Synergy HT multi-mode microplate reader (BioTek, Winooski, VT) and a Bradford assay kit (Bio-Rad, Hercules, CA) with bovine serum albumin as a standard. Protein samples (30 μg for cells, 80 μg for tissues) were run on NuPAGE 4–12% Bis-Tris Gels (Life Technologies, Waltham, MA). The transferred membranes were blocked for 1 h at room temperature, and incubated with primary antibodies (diluted 1:1000) overnight at 4°C. The following polyclonal antibodies were used: rabbit anti-FASN (Novus Biologicals, Littleton, CO), rabbit anti-ACC, rabbit anti phospho-ACC (Cell Signaling Technologies, Danvers, MA), rabbit anti- ACLY (LSBio, Seattle, WA), mouse anti-HSP70 (ThermoFisher Scientific, Waltham, MA), rabbit anti-HSP90 (Thermo Fisher Scientific, Waltham, MA), goat polyclonal anti-HSP60 (Santa Cruz Biotechnology, Dallas, TX), and rabbit anti-GAPDH as housekeeping protein (Santa Cruz Biotechnology, Dallas, TX). The secondary HRP conjugated antibody, at a dilution of 1:5000, was used for 1 h at room temperature. Pre-stained molecular weight marker (Precision Plus Protein Dual Color) was used as a standard (BioRad, Hercules, CA). The signal was visualized by enhanced chemiluminescence (ECL plus; GE Healthcare Bio-Sciences, Buckinghamshire, UK) and captured by FluorChem M MultiFluor System (Proteinsimple, Santa Clara, CA). Image Acquisition and Analysis were performed by AlphaView software (Version 3.4.0, 1993–2011, Proteinsimple, Santa Clara, CA).

### Immunofluorescence staining

Immunofluorescence staining was performed as previously described (Dridi et al., 2012). Briefly, cells were grown in chamber slides and fixed with methanol for 20 min at −20°C. Cells were blocked with serum-free protein block (Dako, Carpinteria, CA) for 1 h at room temperature, and then incubated with primary antibodies (1:200, in Antibody Diluent, Dako, Carpinteria, CA) overnight at 4°C. The signal was visualized with DyLight 488- or 590-conjugated secondary antibody (Thermo Fisher Scientific, Grand Island, NY). Slides were cover slipped with a Vectashield with DAPI (Vector Laboratories, Burlingame, CA), and images were obtained and analyzed using Zeiss Imager M2 and AxioVision software (Carl Zeiss Microscopy).

### Plasma metabolite and hormone measurement

Plasma glucose, triglyceride, cholesterol, lactate dehydrogenase (LDH), and uric acid levels were measured using an automated spectrophotometer as we previously described (Nguyen et al., 2015). Plasma levels of total 3, 5, 3′-triiodothyronine (T_3_) and thyroxine (T_4_) were determined using coated tube radioimmunoassay kits (MP Biomedicals, Solon, OH) as we previously described (Rajaei-Sharifabadi H. et al., 2017).

### Statistical analysis

Growth performance (feed intake and body weight) data were analyzed by two-way repeated measures ANOVA. The rest of the data from both *in vivo* and *in vitro* experiments were analyzed by two-way ANOVA with diet (Control vs. Noni or QCT) and ambient temperature (TN vs. HS) as the main effects. If ANOVA revealed significant effects the means were compared by Tukey's multiple comparison test using GraphPad Prism version 6.00 for Windows, GraphPad Software (La Jolla, CA). Differences were considered significant at *P* < 0.05.

## Results

### Growth performance

**Figure 1:**
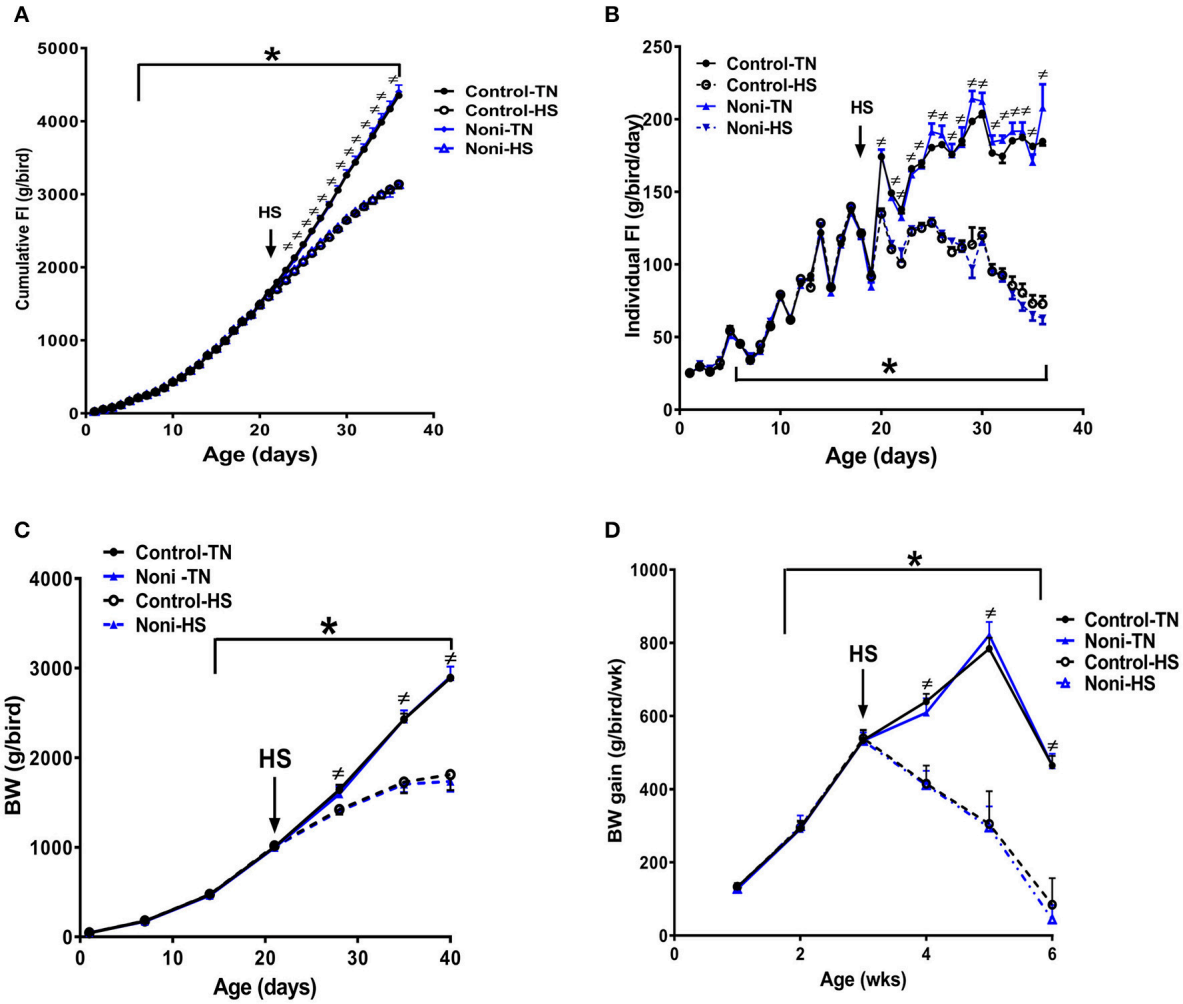
Effects of HS and Noni-enriched diet on feed intake and growth in broilers. HS depressed cumulative **(A)** and individual **(B)** feed intake which in turn results in a reduction of body weight (BW, **C**), and BW gain **(D)**. Data are presented as mean ±SEM (*n* = 160). ≠ denote significant difference between TN and HS conditions at *P* < 0.05. ^*^Indicate significant effect of time at *P* < 0.05.

As depicted in Figure 1, there was no difference between all groups in daily individual or cumulative feed intake (Figures 1A,B) as well as in weekly BW and BW gain during the first 3 weeks of the experiment (Figures 1C,D). However, shortly after heat stress exposure (2 h), core body temperature was significantly increased by ~1°C in control group and Noni supplementation not only delayed this increase but also reduced it (Rajaei-Sharifabadi L. E. H. et al., 2017). During the acute HS exposure, feed intake was significantly reduced by 10% in control diet and 13% in Noni-fed group compared to their counterparts maintained under thermoneutral (TN) conditions (Rajaei-Sharifabadi L. E. H. et al., 2017). This reduction in feed intake was increased over the extended period of HS to reach 28 and 29% after 3 weeks of HS in control diet- and Noni-fed groups, respectively, compared to their counterparts maintained under TN conditions (Figures 1A,B). This, in turn, resulted in a significant reduction in BW by 38% in control diet and 40% in Noni-fed heat stressed groups compared to their TN counterparts (Figures 1C,D).

### Metabolites and thyroid hormone

Acute HS did not affect the plasma levels of all measured parameters (Rajaei-Sharifabadi L. E. H. et al., 2017), however chronic HS significantly increased plasma glucose and uric acid levels (Table 2), and significantly decreased that of T3 and T4 (Table 2). Plasma triglyceride, cholesterol, and LDH levels were not affected by HS (Table 2). Noni supplementation did not elicit any changes to these parameters (Table 2).

**Table 2:** Effects of Noni supplementation and HS on circulating metabolite and hormone levels in broiler chickens.

**Parameters^3^**	**Experimental groups^1^**				***P*****-value^2^**		
	**TN-C**	**TN-N**	**HS-C**	**HS-N**	**D. effect**	**E. effect**	**Interaction**
**Chronic HS**							
*Glc (mg/dL)*	272.75 ± 4.7^a^	274.5 ± 10.2^a^	330.37 ± 11^b^	343.85 ± 10.7^b^	0.52	<0.0001	0.62
*Chol (mg/dL)*	144.50 ± 11^a^	145.5 ± 1.8^a^	188.37 ± 16^b^	192.85 ± 19^b^	0.88	0.02	0.92
*TG (mg/dL)*	35.50 ± 2.7^a^	51 ± 5.1^a^	42.6 ± 7^a^	52 ± 17^a^	0.36	0.76	0.82
*LDH (U/L)*	1795 ± 196^a^	4945 ± 1881^a^	2622.5 ± 870^a^	4562.8 ± 2530^a^	0.22	0.91	0.76
*UA (U/L)*	5.02 ± 1.1^a^	5.25 ± 0.4^a^	9.96 ± 1.3^ab^	14.8 ± 1.9^b^	0.15	0.0004	0.19
*T3 (ng/mL)*	1.65 ± 0.2^a^	1.53 ± 0.06^a^	0.88 ± 0.07^b^	0.818 ± 0.1^b^	0.39	<0.0001	0.81
*T4 (ng/mL)*	14.6 ± 2^a^	12.03 ± 0.5^ab^	9.73 ± 0.8^c^	9.60 ± 0.8^bc^	0.23	0.003	0.27

1 *TN-C, thermoneutral-control diet; TN-N, thermoneutral-Noni diet; HS-C, heat stress-control diet; HS-N, heat stress-Noni diet*.

2 *D, diet; E, environment. Different letters indicate significant difference at P < 0.05*.

3 *Glc, glucose; Chol, cholesterol; TG, triglycerides; LDH, lactate dehydrogenase; UA, uric acid*.

### Heat stress and Noni modulate hepatic HSP expressions in a time-specific manner

**Figure 2:**
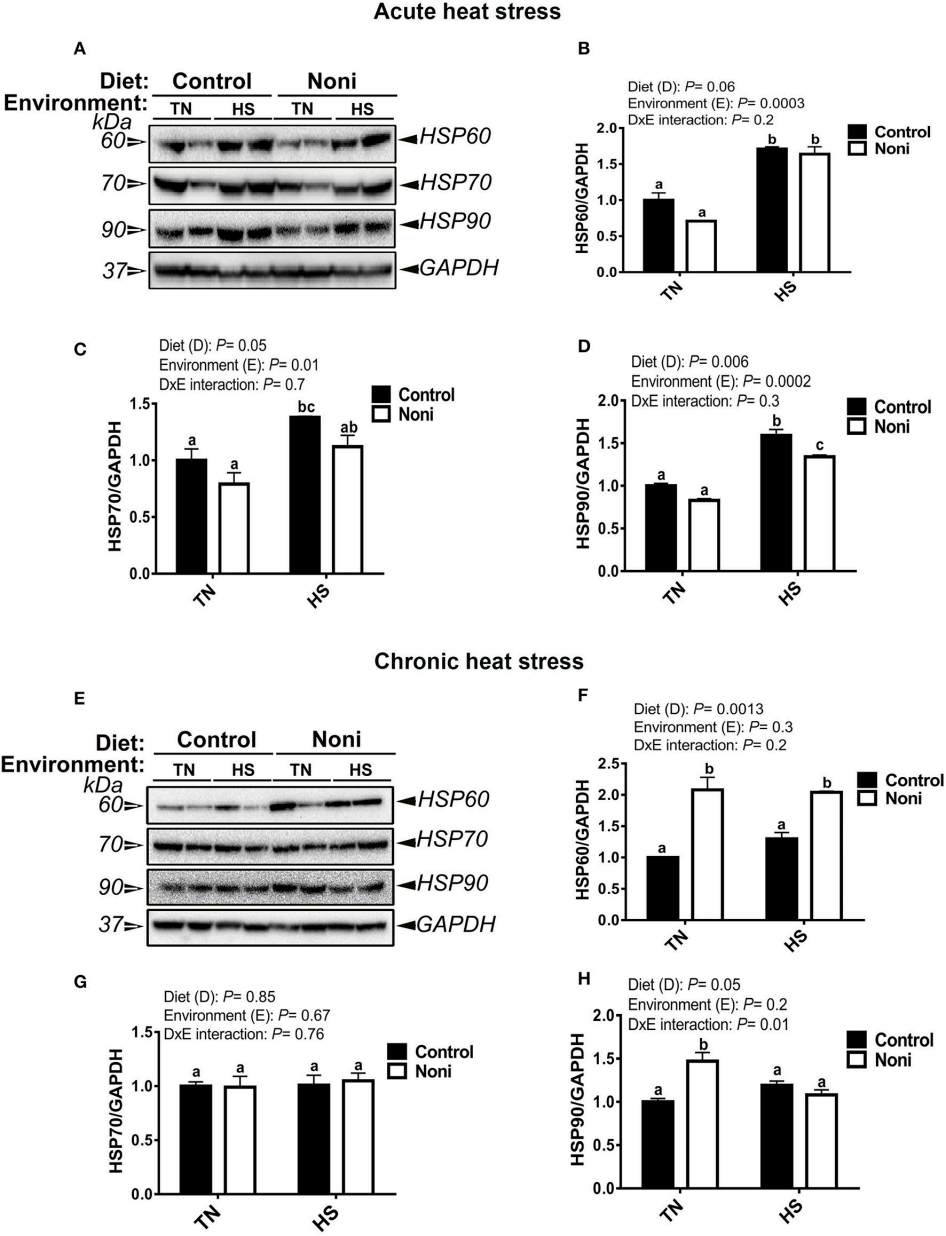
Effect of Noni supplementation on hepatic HSP expression in heat-stressed broilers. **(A–D)** acute HS and **(E–H)** chronic HS. HSP levels were determined by Western blot and their relative expression was presented as a normalized ratio to housekeeping GAPDH protein. Data are presented as mean ± SEM (*n* = 8/group). Different letters indicate significant difference at *P* < 0.05.

As shown in Figure 2, the hepatic expression of HSP60, HSP70, and HSP90 was significantly up-regulated after acute, but not after chronic heat stress (Figures 2A–H). During the acute experimental conditions and in comparison with the control diet, Noni supplementation did not affect the expression of all tested HSPs in the liver of TN group (Figures 2A–D), however it did down-regulate that of HSP90 in the HS group (Figure 2D). Under the chronic experimental conditions, Noni supplementation increased the hepatic expression of HSP60 in both TN and HS groups (Figures 2E,F) and that of HSP90 in the TN group only (Figure 2H). The hepatic expression of HSP70 remained unchanged between the control and Noni groups under both environmental (TN and HS) conditions (Figures 2E,G).

### Heat stress and Noni supplementation differentially regulates the hepatic expression of lipogenic genes and proteins

**Figure 3:**
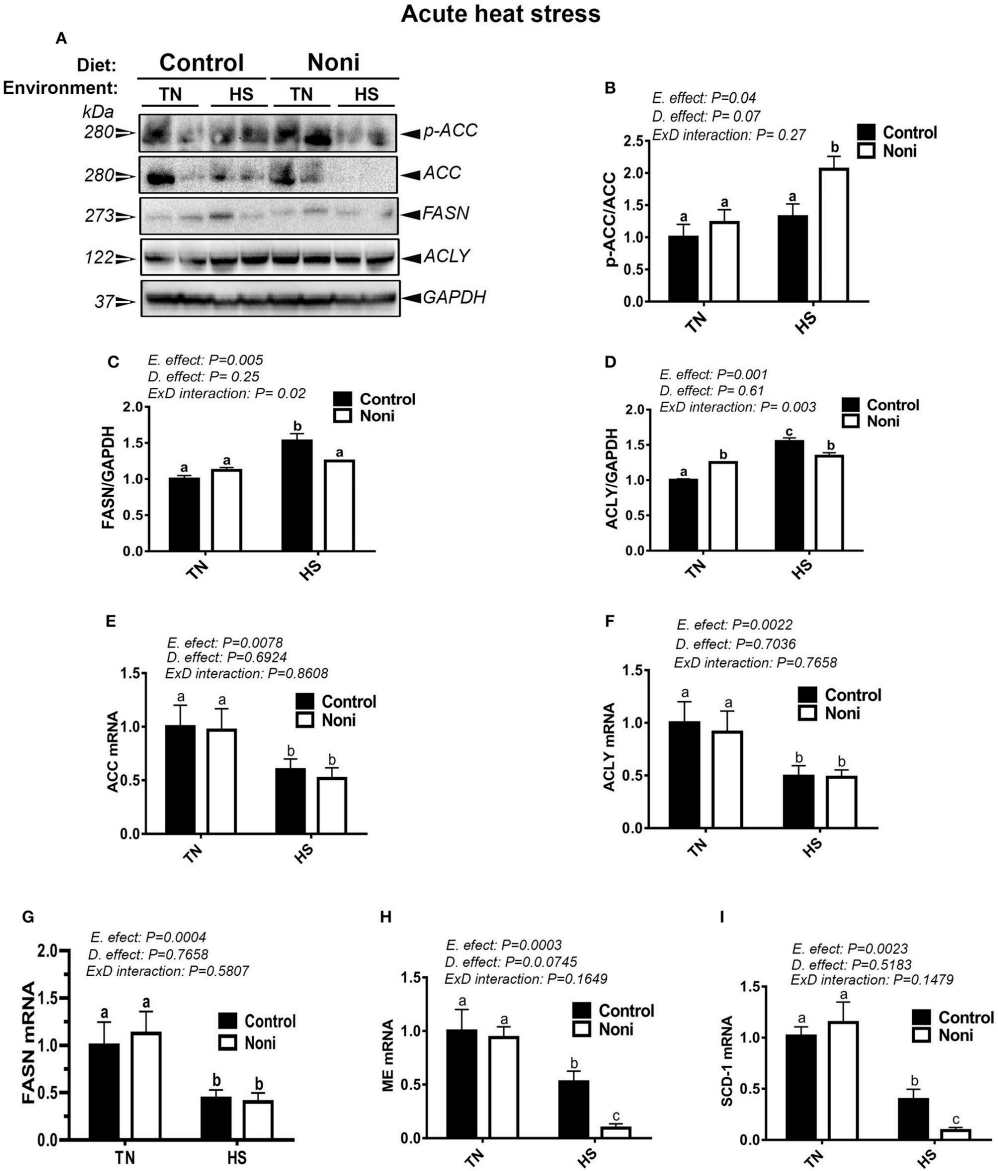
Effect of Noni supplementation on hepatic expression of lipogenic markers in acute heat-stressed broilers. Phosphorylated and pan levels of ACCα as well as FASN and ACLY were determined using Western blot **(A)** and their relative expression was presented as normalized ratio of phosphorylated/total target protein **(B–D)**. Relative abundance of ACCα **(E)**, ACLY **(F)**, FASN **(G)**, ME **(H)**, and SCD-1 **(I)** mRNA was measured by real-time RT-PCR. Data are presented as mean ± SEM (*n* = 8/group). Different letters indicate significant difference at *P* < 0.05.

Under TN conditions and prior to HS, Noni supplementation significantly increased the protein levels of hepatic ACLY, but not that of ACCα and FASN compared to the control diet-fed group (Figures 3A–D). However, the mRNA abundances of these genes remained unchanged (Figures 3E–I). During acute HS, Noni supplementation significantly increased the protein levels of hepatic ACCα, but reduced that of FASN, and ACLY as well as the gene expression of hepatic ME and SCD-1 compared to the control diet-fed group (Figures 3A–I). Interestingly, when all groups were pooled together, acute HS significantly up-regulated the hepatic expression of ACCα, ACLY, and FASN, but it down-regulated the hepatic expression of all tested genes compared to TN group (Figures 3E–I). The induction of FASN and ACLY protein was more noticeable in the heat-stressed birds fed with control diet resulting in a significant interaction (*P* = 0.02 for FASN, *P* = 0.003 for ACLY) between environment and diet (Figures 3B–D).

**Figure 4:**
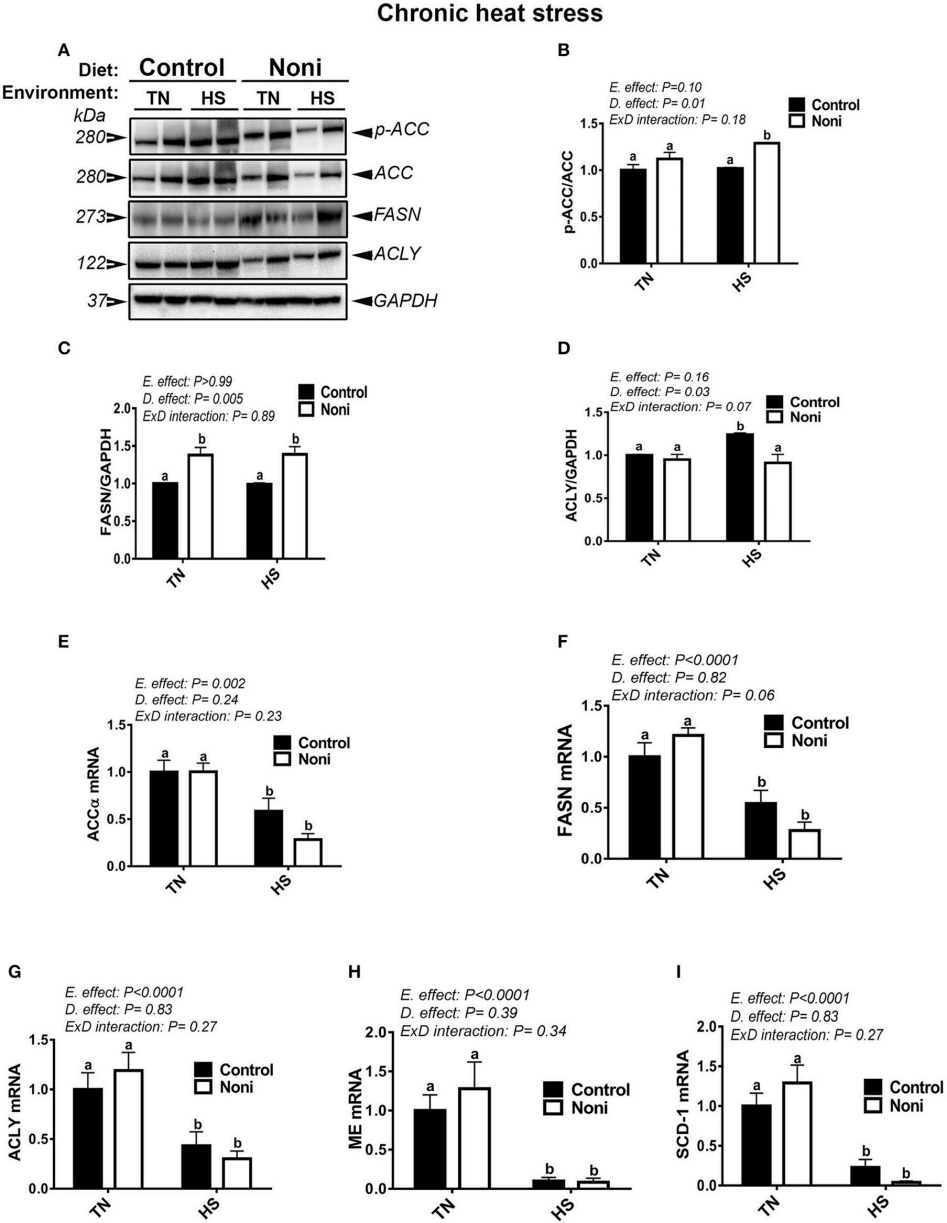
Effect of Noni supplementation on hepatic expression of lipogenic markers in chronic heat-stressed broilers. Phosphorylated and pan levels of ACCα as well as FASN and ACLY were determined using Western blot **(A)** and their relative expression was presented as normalized ratio of phosphorylated/total target protein **(B–D)**. Relative abundance of ACCα **(E)**, ACLY **(F)**, FASN **(G)**, ME **(H)**, and SCD-1 **(I)** mRNA was measured by real-time RT-PCR. Data are presented as mean ± SEM (*n* = 8/group). Different letters indicate significant difference at *P* < 0.05.

Similarly, during the chronic experimental conditions and prior to HS, Noni supplementation did not affect the mRNA abundance of all tested genes (Figures 4E–I) as well as that of hepatic ACCα, and ACLY protein (Figures 4A,B,D). However, the expression of FASN protein was significantly up-regulated in Noni compared to control group (Figure 4C). During chronic HS, Noni supplementation significantly increased the protein levels of hepatic ACCα and FASN, but decreased that of ACLY compared to control diet-fed group (Figures 4B–D). The mRNA levels of all tested genes did not differ between Noni- and control diet-fed groups reared under chronic HS (Figures 4E–I). Interestingly, when all groups were pooled together, chronic HS significantly down-regulated the hepatic expression of all tested genes (Figures 4A–I).

### Heat stress modulates the hepatic expression of lipogenesis-related transcription factors

**Figure 5:**
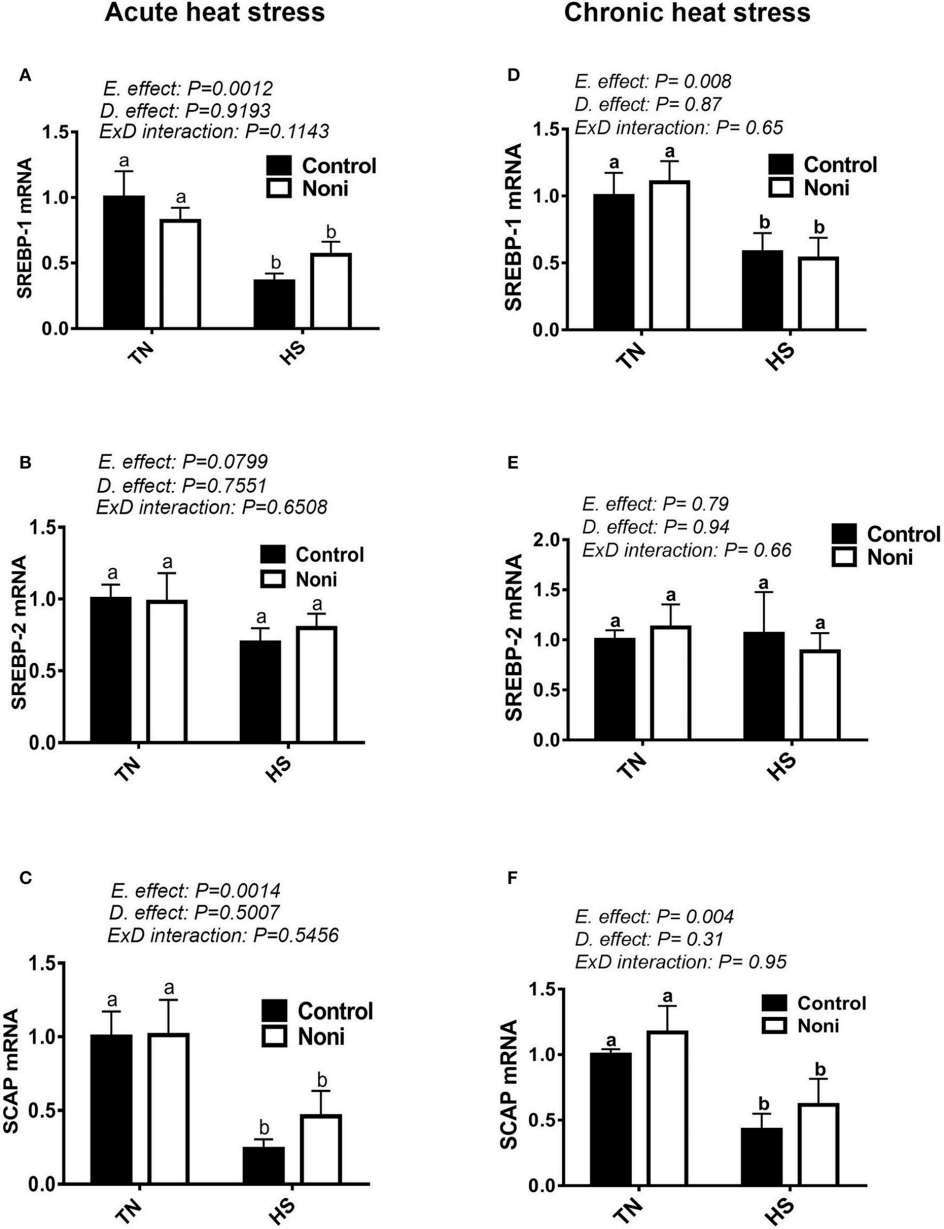
Effect of Noni on hepatic expression of lipogenesis-related transcription factors in heat stressed broilers. **(A–C)** acute HS and **(D–F)** chronic HS. Relative expression of SREBP-1 **(A,D)**, SREBP-2 **(B,E)**, and SCAP **(C,F)** was measured by real-time RT-PCR. Data are presented as mean ± SEM. D, diet; E, environment; TN, thermoneutral; HS, heat stress. Different letters indicate significant difference at *p* < 0.05.

As shown in Figure 5, both acute and chronic HS significantly down-regulated the hepatic expression of SREBP-1 and SCAP gene (Figures 5A,C,D,F), however it did not affect that of SREBP-2 (Figures 5B,E), INSIG-2, PPARα, or PPARγ (Table 3). Noni supplementation did not elicit any change to the expression of these transcription factors under both environmental (TN and HS) conditions (Figures 5A–F).

**Table 3:** Effects of Noni supplementation on hepatic expression of INSIG2, PPARα and PPARγ genes in heat stressed broiler chickens.

**Parameters^3^**	**Experimental groups^1^**				***P*****-value^2^**		
	**TN-C**	**TN-N**	**HS-C**	**HS-N**	**D. effect**	**E. effect**	**Interaction**
**ACUTE HS**							
*INSIG2*	1 ± 0.2	0.60 ± 0.06	0.62 ± 0.09	0.97 ± 0.2	0.91	0.99	0.10
*PPARα*	1 ± 0.3	0.57 ± 0.02	1.11 ± 0.1	1.21 ± 0.3	0.65	0.30	0.47
*PPARγ*	1 ± 0.3	0.74 ± 0.1	0.83 ± 0.2	1.24 ± 0.3	0.82	0.64	0.36
**CHRONIC HS**							
*INSIG2*	1 ± 0.1	0.63 ± 0.1	0.92 ± 0.1	0.90 ± 0.1	0.26	0.55	0.29
*PPARα*	1 ± 0.3	0.58 ± 0.1	0.97 ± 0.1	0.87 ± 0.2	0.32	0.61	0.54
*PPARγ*	1 ± 0.3	0.43 ± 0.1	1.51 ± 0.3	1.58 ± 0.8	0.73	0.26	0.66

*Relative abundance of INSIG2, PPARα, and PPARγ mRNA was measured by qPCR. Data are presented as mean ± SEM (n = 8/group)*.

1 *TN-C, thermoneutral-control diet; TN-N, thermoneutral-Noni diet; HS-C, heat stress-control diet; HS-N, heat stress-Noni diet*.

2 *D, diet; E, environment*.

3 *INSIG2, insulin induced gene 2; PPARα/γ, peroxisome proliferator activated receptor alpha/gamma*.

### Heat stress modulates the hepatic expression of lipolytic genes

**Figure 6:**
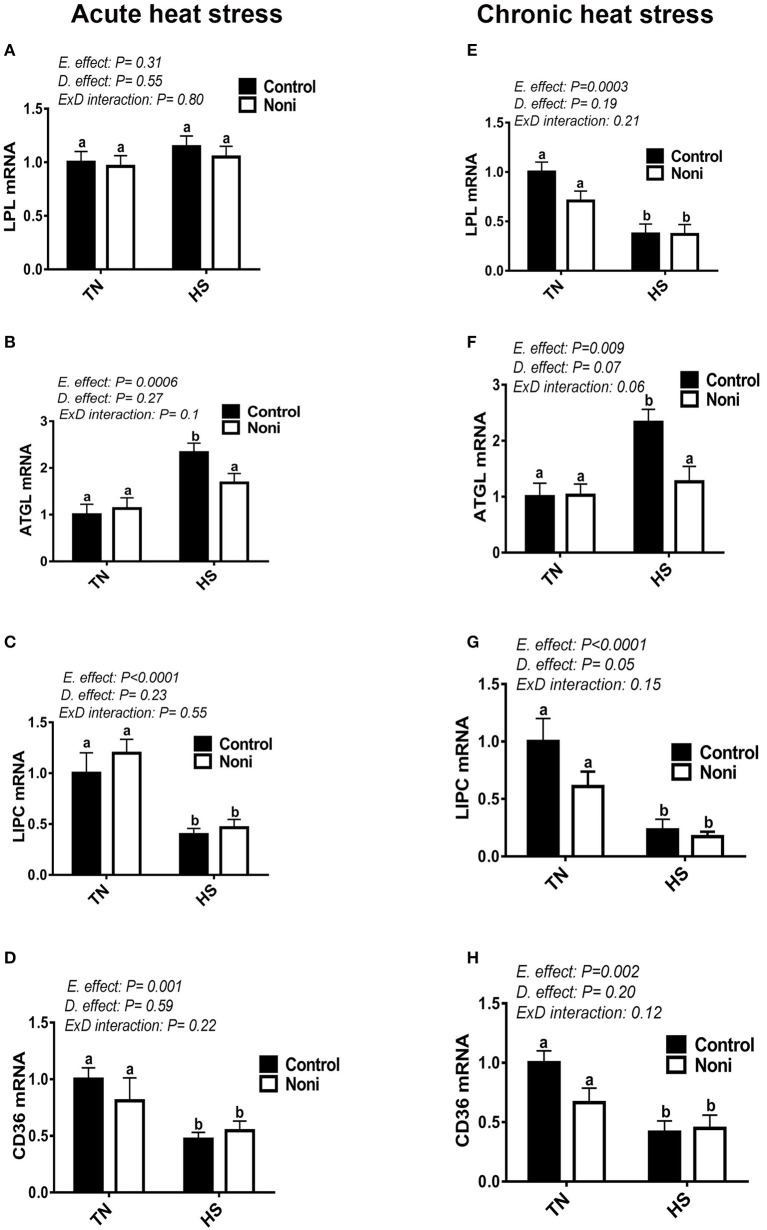
Effect of Noni supplementation on hepatic expression of lipolytic pathway in heat-stressed broilers. **(A–D)** acute HS, **(E–H)** chronic HS. Relative expression of LPL **(A,E)**, ATGL **(B,F)**, LIPC **(C,G)**, and CD36 gene **(D,H)** was measured by real-time RT-PCR. Data are presented as mean ± SEM (n = 8/group). Different letters indicate significant difference at P < 0.05.

In comparison with the control diet, Noni supplementation did not affect the hepatic expression of LPL, ATGL, LIPC, and CD36 genes under the TN environment for both acute and chronic experimental conditions (Figures 6A–H). Under HS exposure, however, Noni significantly decreased the mRNA levels of hepatic ATGL in both acute and chronic experimental conditions (Figures 6B,F). When all groups were pooled together, both acute and chronic HS down-regulated the hepatic expression of LIPC and CD36 gene compared to TN conditions (Figures 6C,D,G,H). However, the hepatic LPL mRNA levels were significantly decreased only after chronic but not acute HS (Figures 6A,E). Interestingly, both acute and chronic HS up-regulated the hepatic expression of ATGL gene compared to TN condition (Figures 6B,F) and this increase was more pronounced in the control diet-fed group.

### QCT, the active ingredient of Noni, modulates lipogenic gene expression in sim-CEL cells

**Figure 7:**
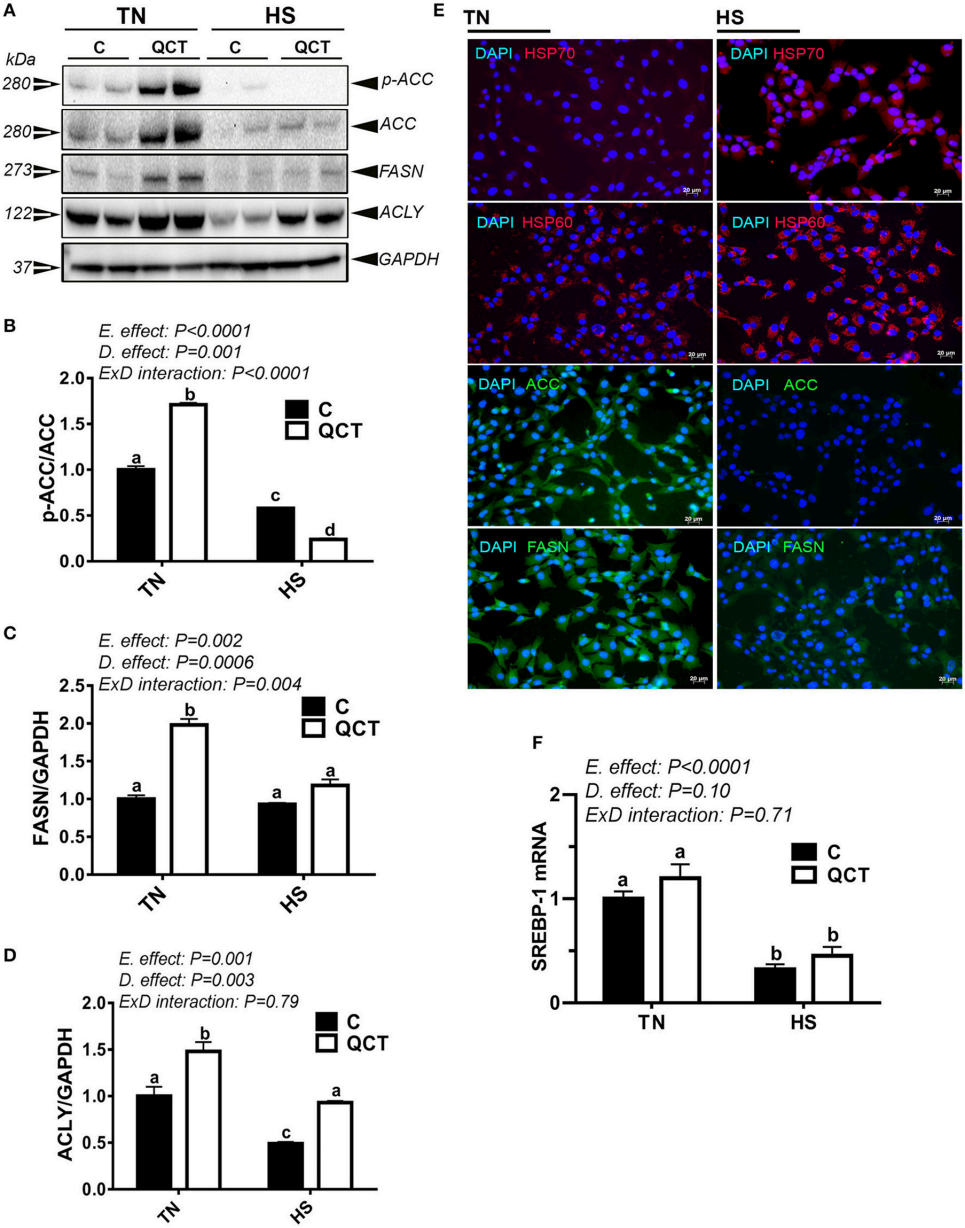
Effect of QCT on lipogenesis-related signatures and SREBP-1 gene expression in heat stressed sim-CEL cells. Phosphorylated and pan levels of ACCα as well as FASN and ACLY were determined using Western blot and their relative expression was presented as normalized ratio of phosphorylated/total target protein **(A–D)**. Immunofluorescence staining **(E)** shows that HS induce HSP 60 and 70, and reduces ACCα and FASN protein levels in sim-CEL cells. SREBP-1 mRNA abundance was measured by real-time RT-PCR **(F)**. Data are presented as mean ±SEM (n = 6/groups). Different letters indicate significant differences at P < 0.05. Western blotting figure is representative of 3 replicates.

HS significantly down-regulated the expression of lipogenic genes at mRNA and protein levels (Figures 7A–E, Table 4) as well as their related transcription factors SREBP-1, SREBP-2, and SCAP (Figure 7F, Table 4). However, HS did not alter the expression of LPL, ATGL, and LIPC (Table 4). The up-regulation of both HSP60 and HSP70, demonstrated by immunofluorescence staining (Figure 7E), indicated that sim-CEL cells were undergoing stress induced by heat load. QCT treatment significantly increased ACCα, FASN, and ACLY protein levels in control (37°C) environment only, however it did significantly reduced the phosphorylated levels of ACCα at Serine 79 site and increased that of ACLY protein levels under HS conditions (Figures 7B,D).

**Table 4:** Effects of quercetin (QCT) supplementation on hepatic expression of lipogenic-lipolytic genes in heat stressed Celi cells.

**Parameters^3^**	**Experimental groups^1^**				**P-value^2^**		
	**C-37°C**	**QCT-37°C**	**C-45°C**	**QCT-45°C**	**D. effect**	**E. effect**	**Interaction**
ACCα	1 ± 0.08^a^	1.16 ± 0.08^a^	0.61 ± 0.08^b^	0.65 ± 0.1^b^	0.36	0.0032	0.56
ACLY	1 ± 0.07^a^	0.04^a^	0.37 ± 0.03^b^	0.36 ± 0.05^b^	0.30	<0.0001	0.39
FASN	1 ± 0.1^a^	0.91 ± 0.1^a^	0.19 ± 0.03^b^	0.26 ± 0.1^b^	0.94	<0.0001	0.49
SCD-1	1 ± 0.06^a^	1.38 ± 0.1^a^	0.15 ± 0.01^b^	0.20 ± 0.03^b^	0.02	<0.0001	0.06
SREBP-2	1 ± 0.05^a^	1.11 ± 0.08^a^	0.34 ± 0.02^b^	0.45 ± 0.04^b^	0.09	<0.0001	0.98
SCAP	1 ± 0.07^a^	1.36 ± 0.1^a^	0.24 ± 0.05^b^	0.39 ± 0.05^b^	<0.0001	0.01	0.21
LPL	1 ± 0.3^a^	1.45 ± 0.4^a^	1.08 ± 0.2^a^	1.59 ± 0.5^a^	0.32	0.8	0.95
ATGL	1 ± 0.1^a^	1.91 ± 0.4^a^	1.66 ± 0.2^a^	1.80 ± 0.2^a^	0.09	0.34	0.20
LIPC	1 ± 0.2^a^	0.80 ± 0.2^a^	0.45 ± 0.07^b^	0.70 ± 0.1^a^	0.90	0.16	0.32

*Relative abundance of ACCα, ACLY, FASN, SCD-1, SREBP-2, SCAP, LPL, ATGL, and LIPC mRNA was measured by qPCR. Data are presented as mean ± SEM (n = 8/group)*.

1 *C, Control; QCT, quercetin*.

2 *D, diet; E, environment*.

3 *AACα, acetyl-CoA carboxylase alpha; ACLY, ATP citrate lyase; FASN, fatty acid synthase; SCD-1, stearoyl-CoA desaturase 1; SREBP-1/2, sterol regulatory element-binding protein 1/2; SREBP cleavage-activating protein; LPL, lipoprotein lipase; ATGL, adipose triglyceride lipase; LIPC, hepatic triacylglycerol lipase*.

*Different letters indicate significant differences at p < 0.05*.

## Discussion

HS adversely affects poultry production through depressed feed intake and growth as shown in many previous reports (Dale and Fuller, 1980; Cahaner and Leenstra, 1992; Leenstra and Cahaner, 1992; Deeb et al., 2002) and confirmed by the present study. These effects are mediated by numerous molecular, neuroendocrine, and physiological processes including nutrient portioning and lipid metabolism that are not completely defined, at least, in avian species.

As commercial broilers are fed lipid-poor diets (< 10%), the majority of the accumulated fat is derived from the liver which is the main site for lipogenesis (Goodridge and Ball, 1967; Leveille et al., 1968; Yeh and Leveille, 1971). Indeed, the avian liver is responsible for more than 90% of de novo fatty acid synthesis and is also a site of lipid storage in birds (Goodridge and Ball, 1967; Leveille et al., 1968; Yeh and Leveille, 1971). The increased levels of hepatic FASN and ACLY proteins, rate limiting enzymes in de novo fatty acid synthesis, indicates that HS induce hepatic lipogenesis in chickens which corroborates previous study reported by Geraert et al. (1996) and Jastrebski et al. (2017). However, the upregulation of p-ACCα^Ser79^ by HS, which indicates that the enzyme is less active, is intriguing. Although it is not clear at this time-point why ACCα is down-regulated, it is possible that a compensatory increase in another ACC isoform such as, ACCβ (ACC2) is involved. Furthermore, as its regulation is complex, it is possible that the down regulation of ACCα might be due to a negative feedback loop. For instance, the ACCα decrease could result from an increase in glucagon and/or catecholamine levels which has been reported to be induced by stressful stimuli including HS and feed deprivation (Bloom et al., 1973; Iguchi et al., 2012). Although we didn't determine the plasma glucagon levels in the present study, the significant increase in circulating glucose concentration (23%) by HS, despite the significant reduction in feed intake, supports the above mentioned hypothesis. In fact, during HS along with reduced energy intake, glucagon plays a key role in maintaining glucose homeostasis via stimulating hepatic glycogenolysis and gluconeogenesis (Exton and Park, 1968; Garrison and Haynes, 1973; Watford, 1985) which is evidenced in our study by the upregulation of hepatic phosphoenolpyruvate carboxykinase (PEPCK), glycogene phosphorylase (GLGP), and fructose 2,6-biphosphatase 4 (PFKFB4) (data not shown). An additional possibility is that HS activates the energy sensor AMPKα1/2 (data not shown) and AMPK has been shown to phosphorylate ACCα^Ser79^ (Park et al., 2002). We should note that we measured here only one ACC phosphorylation site (Ser79), however there are several other sites including Ser1200 and Ser1215 (Brownsey et al., 2006) that might be differently regulated and phosphorylated by HS.

HS down regulated the hepatic expression of all tested lipogenic genes as well as their related transcription factors which is different from previous integrated transcriptomic studies reported in rodents and chickens (Bhusari et al., 2007; Jastrebski et al., 2017). This discrepancy might due to the technique used and the choice of the normalization (housekeeping) control (real-time qPCR in our study vs. RNAseq or microarray in previous studies) and/or experimental conditions (species, age, duration, intensity, and/or severity of HS, diet composition, etc.). The absence of correlation between gene and protein expression in our study indicates that the transcription and translation of these target markers are differentially regulated by HS which is not surprising. It is plausible that under our experimental stress conditions, the pool of already synthesized lipogenic mRNA might be efficiently translated with a quick turnover or degradation, however its protein product accumulation increases and remain in the cellular pool due to its high half-life (Schwanhausser et al., 2011). The protein stability may also increase by post-translational modification like acetylation or glycosylation. An additional possibility is that mRNA translation can be upregulated by RNA binding proteins and/or down-regulation of micro (mi) RNA targeting that mRNA.

Interestingly, our study showed that the lipolytic pathway is also expressed in the chicken liver which is a considerable site for lipid storage and secretion (Hermier, 1997). HS seems to regulate the expression of hepatic lipolysis-related genes in a time-specific manner. For instance, LPL mRNA abundance was not affected by acute HS, but it was decreased by chronic HS. Both acute and chronic HS up regulate hepatic ATGL, but decreased LIPC expression which may explain the unchanged levels of plasma triglycerides. As LPL has other functions beside its hydrolytic activity, it is conceivable that HS might affect the maturation of plasma HDL (Strauss et al., 2001), uptake of lipoprotein-associated lipids and vitamins (Rinninger et al., 1998, 2001; Goti et al., 2002), and/or degradation of lipoprotein (Heeren et al., 2002) which warrant further investigations. Of particular interest, a novel function of LPL in the regulation of appetite and energy balance regulation has recently been postulated (Wang et al., 2011). Thus, it is possible that the reduced feed intake and body weight induced by HS in this study might be mediated via down regulation of LPL expression and this merit further in depth studies.

As expected and in agreement with previous studies (Xie et al., 2014; Wang et al., 2015), HSPs (HSP60, HSP70, and HSP90) were all upregulated by acute HS, but remained unchanged after longer HS exposure compared to TN conditions. This later observation is not surprising and indicates that the birds become acclimated to chronic HS which in turn results in no further increase in cellular HSPs (Givisiez et al., 1999). Noni supplementation reduced the expression of HSP90, but not that of HSP60 and HSP70-induced by acute HS suggesting that the regulation of HSP by Noni is family (molecular weight)-dependent. However, contrary to our expectation, Noni-enriched diet upregulated the hepatic expression of HSP60 and HSP90 in broilers maintained under TN conditions, as well as that of HSP60 in chronic heat-stressed broilers without affecting the expression of HSP70. The down regulation of hepatic HSP90 during acute HS supports the notion that Noni might alleviate stress induced by heat load and this has been substantiated by the reduction of core body temperature-induced by HS (data not shown). However, the upregulation of both HSP60 and HSP90 during the chronic but not acute TN conditions is intriguing. It has been reported that Noni juice induced stress and reduced body weight in mice (Muto et al., 2010). This is an unlikely scenario in our experimental conditions because birds fed control or Noni diet showed similar feed intake, body weight, and body weight gain without any sign of discomfort or stress (physical activity and behavior). In addition, necropsy analysis of all major organs in both groups revealed no pathology. Although the physiological meaning and biological determinants are not known at this time point, it is foreseeable that age (3 vs. 6 weeks in acute vs. chronic conditions, respectively) might impact the effect of Noni on the hepatic HSP60 expression. Age-dependent differences in liver gene expression has been recently reported (Uno et al., 2017) and HSP60 levels have been found to progressively decline with increasing age in humans (Rea et al., 2001). Besides their role in stress response, HSPs act also as molecular chaperones under normal physiological conditions where they carry out such essential functions as protein translocation, folding, and assembly (Hendrick and Hartl, 1993). It is, therefore, possible that HSP might mediate the effect of Noni in regulating these cellular processes, however owing to the gap in avian HSP biology, experimental evidence using mechanistic and functional studies supporting these possibilities needs to be provided.

The lipogenic proteins followed a similar hepatic expression pattern as HSPs across our experimental conditions. For instance, HSPs (HSP70 and HSP90), FASN, and ACLY showed similar expression patterns in the liver of acute heat-stressed broilers. Similarly, the hepatic expression of HSP60 and FASN follow the same trend under chronic HS conditions. As HSPs are now understood to be involved in many cellular processes from transcription regulation to cell death signaling (Rutherford and Zuker, 1994), our data suggest that HS might modulate the expression of lipogenic protein through HSPs. Tang and co-workers (Tang et al., 2016) have shown that HSP60 silencing alter directly the expression of FASN and ACCα in glioblastoma cells. FASN has also been recently identified as a bona fide HSP90 client (Fierro-Monti et al., 2013). Zhang et al. (2012) reported that HSP90 binds to AMPK and regulates ACCα expression.

To gain a better understanding of HS responses, we next evaluated the effect of HS alone or in combination with QCT on the same parameters by using chicken hepatocyte cell culture. We chose QCT because it is a highly bioactive ingredient of Noni with an average concentration of 7.4 mg/g (CP Foods, personal communication, Kampkotter et al., 2007). Although the optimal temperature of cell culture (37°C) and chicken body temperature (~41°C) are quite different, HS induced the expression of HSP60 and HSP70 in sim-CEL cells comparably to chicken liver tissues. However, it down-regulated the expression of lipogenic (ACCα, FASN, and ACLY) proteins *in vitro* which is opposite to that in animals. This indicates, firstly, that HS may directly alter the expression of hepatic lipogenic genes independently of the reduction of feed intake. Secondly, the differential expression profile of hepatic lipogenic proteins between the *in vivo* and *in vitro* HS studies might be due to the involvement of other organs such as, the brain (central neural pathways for thermoregulation) (Morrison and Nakamura, 2011) and the hormonal milieu. In fact, we have previously shown that HS altered the hepatic expression of leptin (Dridi et al., 2008) which is a key regulator of lipogenic genes (Dridi et al., 2005). Additionally, HS has been shown to dysregulate several other appetite-related hormones including ghrelin, thyroid hormone, and adipocytokines (i.e., adiponectin) and these hormones orchestrate the regulation of hepatic lipogenic genes (Yin et al., 2005; Tao et al., 2006; Buyse et al., 2009; Morera et al., 2012). QCT treatment induces the expression of lipogenic protein in sim-CEL cells maintained under both control and short (4 h) HS exposure, indicating that a short-term control mechanism of lipogenesis by QCT is effective. Moreover, the prompt responsiveness of cultured hepatocytes to QCT suggest that the QCT effect may be direct rather than secondary. The disaccord of QCT effects between chicken and mammalian hepatocytes is striking. It has been reported that QCT administration inhibits FASN expression in human HepG2 (Li et al., 2013; Zhao et al., 2014), rat H4llE (Zhou et al., 2014), and rat BRL-3A cells (Wang et al., 2013). This discrepancy might be due to dose and/or species-specific regulation of lipogenic genes by QCT (Dridi et al., 2006). In fact, QCT has been shown to have a dose-dependent biphasic effect on mammalian cell proliferation (van der Woude et al., 2003) and on CYP1A2 expression (Kang et al., 2004).

**Figure 8:**
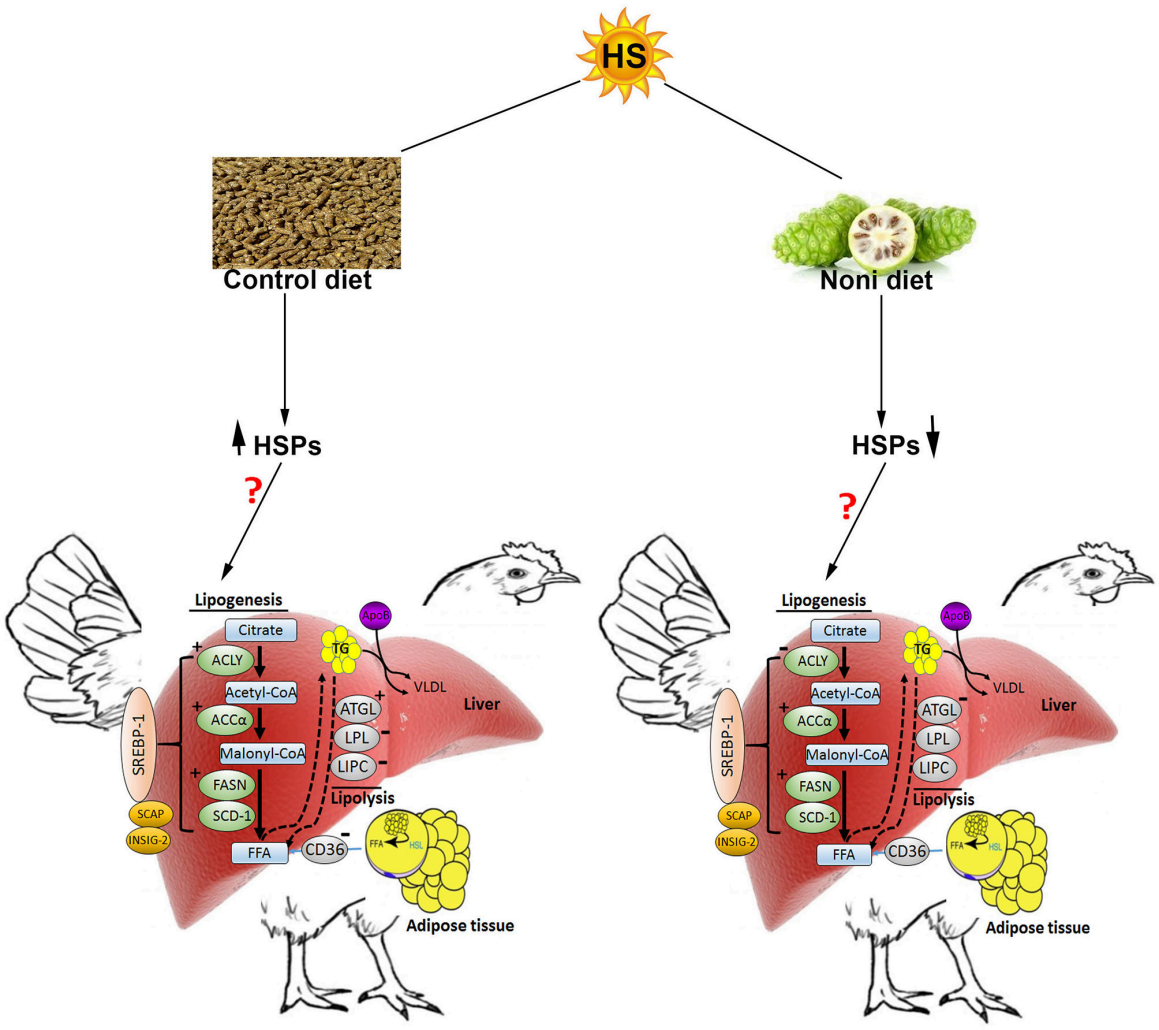
Schematic representation of the effect of heat stress and Noni supplementation on hepatic lipid metabolism in broilers (from protein expression analysis). Heat stress up-regulated the hepatic expression of heat shock proteins (HSP60, HSP70, and HSP90) as well as key lipogenic proteins (FASN, ACCα, and ACLY). HS down-regulated the hepatic expression of LPL and LIPC, but up-regulated ATGL. Noni supplementation down-regulated the hepatic expression of HSPs, ACCα, and FASN but it down regulated ACLY. ↑ and “+” denote induction, “−“ indicates inhibition. HSL, hormone sensitive lipase; FFA, free fatty acids; VLDL, very low density lipoprotein.

In conclusion (Figure 8), HS induces the hepatic expression of HSPs and stimulates *de novo* fatty acid synthesis in broilers which may explain the increased fat deposition observed during hot season (Geraert et al., 1996). In this study, Noni supplementation did not ameliorate feed intake and growth depression under HS conditions, but it did modulate the hepatic expression of HSPs in a time-specific manner suggesting a potential role in stress response that merit further investigations. Possibly, the lack of effect of Noni in the present study could be attributed to clean litter and low relative humidity (20%) in the experimental environmental chambers. A less pristine environment such as, that found in commercial poultry houses may be required to demonstrate potential benefits of Noni.

## Author contributions

JF, HR-S, and EG performed the experiments. LB and BH generate the immunofluorescence data. LE and TP measured the thyroid hormone levels. SD, AD, and WB purchased the reagents. SD designed the experiment and wrote the manuscript.

### Conflict of interest statement

The authors declare that the research was conducted in the absence of any commercial or financial relationships that could be construed as a potential conflict of interest.
